# Full-Length Transcriptome Construction and Systematic Characterization of Virulence Factor-Associated Isoforms in *Vairimorpha* (*Nosema*) *Ceranae*

**DOI:** 10.3390/genes15091111

**Published:** 2024-08-23

**Authors:** Sijia Guo, He Zang, Xiaoyu Liu, Xin Jing, Zhitan Liu, Wende Zhang, Mengyi Wang, Yidi Zheng, Zhengyuan Li, Jianfeng Qiu, Dafu Chen, Tizhen Yan, Rui Guo

**Affiliations:** 1 College of Bee Science and Biomedicine, Fujian Agriculture and Forestry University, Fuzhou 350002, China; guosijia1998@163.com (S.G.); zanghe321@163.com (H.Z.); liuxiaoyu2000@163.com (X.L.); jingxin6662022@163.com (X.J.); 17339838732@163.com (Z.L.); wdebee@163.com (W.Z.); 13277017210@163.com (M.W.); zzz974429@163.com (Y.Z.); lzy109364@163.com (Z.L.); jfqiu@fafu.edu.cn (J.Q.); dfchen826@fafu.edu.cn (D.C.); 2 National & Local United Engineering Laboratory of Natural Biotoxin, Fuzhou 350002, China; 3 Apitherapy Research Institute of Fujian Province, Fuzhou 350002, China; 4 Institute of Reproduction and Genetics, Dongguan Maternal and Children Health Hospital, Dongguan 510110, China

**Keywords:** *Vairimorpha (Nosema) ceranae*, microsporidian, third-generation sequencing, PacBio SMRT, full-length transcriptome, alternative splicing

## Abstract

*Vairimorpha (Nosema) ceranae* is a single-cellular fungus that obligately infects the midgut epithelial cells of adult honeybees, causing bee microsporidiosis and jeopardizing bee health and production. This work aims to construct the full-length transcriptome of *V. ceranae* and conduct a relevant investigation using PacBio single-molecule real-time (SMRT) sequencing technology. Following PacBio SMRT sequencing, 41,950 circular consensus (CCS) were generated, and 25,068 full-length non-chimeric (FLNC) reads were then detected. After polishing, 4387 high-quality, full-length transcripts were gained. There are 778, 2083, 1202, 1559, 1457, 1232, 1702, and 3896 full-length transcripts that could be annotated to COG, GO, KEGG, KOG, Pfam, Swiss-Prot, eggNOG, and Nr databases, respectively. Additionally, 11 alternative splicing (AS) events occurred in 6 genes were identified, including 1 alternative 5′ splice-site and 10 intron retention. The structures of 225 annotated genes in the *V. ceranae* reference genome were optimized, of which 29 genes were extended at both 5′ UTR and 3′ UTR, while 90 and 106 genes were, respectively, extended at the 5′ UTR as well as 3′ UTR. Furthermore, a total of 29 high-confidence lncRNAs were obtained, including 12 sense-lncRNAs, 10 lincRNAs, and 7 antisense-lncRNAs. Taken together, the high-quality, full-length transcriptome of *V. ceranae* was constructed and annotated, the structures of annotated genes in the *V. ceranae* reference genome were improved, and abundant new genes, transcripts, and lncRNAs were discovered. Findings from this current work offer a valuable resource and a crucial foundation for molecular and omics research on *V. ceranae*.

## 1. Introduction

*V. ceranae* is a microsporidian parasite presently known to infect the Asian honey bee, *Apis cerana*, as well as the European honey bee, *Apis mellifera* [1,2]. As an obligate fungal pathogen that infects the midgut epithelial cells of honeybees, *V. ceranae* is infectious to queens, drones, and worker bees [3]. It is spread through the fecal–oral system in the bee colonies, germinating in a specific midgut environment after entering the bee and injecting sporoplasms into the midgut epithelial cells by polar filamentous catapulting. In the midgut epithelial cells, it completes its own metabolism and proliferation with the material energy of the host [4]. The *V. ceranae* infection affects the population and productivity of the bees and causes great loss for the beekeeping industry [5].

As early as 2009, Cornman et al. performed the first complete genome sequencing of *V. ceranae* with the Roche 454 sequencing platform, assembling 5465 contigs with an N50 of 2902 bp [6]. In the past decade, next-generation sequencing (NGS) technology represented by Illumina has been widely used in the genome sequencing of animals [7], plants [8], and microorganisms [9] with the advantages of high throughput and continuous cost reduction, which has greatly improved the quality of genomes and transcriptomes of species. Some transcriptome studies examining the interactions between *V. ceranae* and honeybee hosts have employed NGS [10,11,12,13]. By using Illumina sequencing technology, Pelin et al. again assembled and annotated the genome of *V. ceranae*, including 536 contigs with an N50 of 42,592 bp [14]. However, the GC bias and short reads of NGS need to be spliced using bioinformatic methods, and there are obvious disadvantages in identifying repeat sequences and structural analyses. 

In recent years, with the rapid development and application of third-generation sequencing (TGS) technology represented by PacBio single-molecule real-time (SMRT) sequencing technology and Oxford nanopore long-read sequencing technology, the chromosome-level genomes of an increasing number of species have been assembled based on TGS alone or in combination with NGS [15]. In comparison with genome sequencing, TGS-based transcriptome sequencing has a lower cost and a shorter cycle time. The improvements in the genome annotation of wheat (*Triticum aestivum*) [16] and Ceylon hookworm (*Ancylostoma ceylanicum*) [17] have been reported previously. In addition, TGS also has the advantage of directly reading nucleic acid modifications and simultaneously identifying various isoform forms of the same gene. Previous studies on transcriptome complexity, such as *Trifolium pratense* and *Sus scrofa,* were completed by PacBio SMRT. In the study of *T. pratense*, Chao et al. identified 29,730 novel isoforms from known genes and 2194 novel isoforms from new genes [18]. Li et al. identified a total of 389,781 high-quality full-length non-chimerics (FLNCs) in *S. scrofa*, covering 77,075 isoforms [19]. On the basis of PacBio SMRT sequencing, Chen et al. generated the full-length transcriptome of *Ascosphaera apis*, a widespread fungal pathogen of honeybee larvae that results in chalkbrood disease, which included not only 174,095 highly confident transcripts covering 5141 known genes with an average length of 2728 bp but also 2405 genic loci and 11,623 isoforms that have not been annotated yet within the current reference genome [20].

Currently, a TGS-based transcriptomic investigation regarding *V. ceranae* is missing, hindering a deep understanding of the complexity of the *V. ceranae* transcriptome and related molecular studies. In this work, PacBio SMRT sequencing was employed in collaboration with Illumina sequencing to construct and annotate the full-length transcriptome of *V. ceranae*, followed by the identification of alternative splicing (AS) events and alternative polyadenylation (APA) sites. Furthermore, full-length transcripts relevant to virulence factors, energy metabolism, and material metabolism were investigated and verified. Our results could reveal the complexity of the *V. ceranae* transcriptome, offer a valuable resource for relevant omics and molecular studies, and lay a foundation for further functional dissection of *V. ceranae* isoforms associated with virulence factors and metabolisms.

## 2. Materials and Methods

### 2.1. Bee and Fungus

*Apis mellifera ligustica* colonies were reared in the teaching apiary of the College of Bee Science and Biomedicine, Fujian Agriculture and Forestry University, Fuzhou, China. *V. ceranae* spores were isolated and conserved at the Honey Bee Protection Laboratory of the College of Bee Science and Biomedicine and the China General Microbiological Culture Collection Center (CGMCC NO. 28110).

### 2.2. Preparation of V. ceranae Spores

Newly emerged workers (*n* = 20) were collected, starved for 2 h, and then each fed 5 μL of 50% (*w*/*v*) sucrose solution containing 1 × 10^6^ spores of *V. ceranae* [21] until the entire droplet was consumed [22]. At 14 d post-inoculation (dpi) with *V. ceranae*, the infected workers were first kept at −20 °C for 20 min to anesthetize them, and then, fresh spores of *V. ceranae* were isolated and purified according to the method described by Cornman et al. [6] with some minor modifications. The midguts were separated with clean dissection tweezers, homogenized in distilled water, filtered through four layers of sterile gauze, and then centrifuged three times at 6000× *g* for 5 min. The supernatant was discarded as the spores remained in the sediment, and the resuspended pellet was further purified on a discontinuous Percoll gradient (Solarbio) consisting of 5 mL each of 25%, 50%, 75%, and 100% Percoll solution. The spore suspension was then overlaid onto the gradient and centrifuged at 18,000× *g* for 90 min at 4 °C. The spore pellet was carefully extracted with a sterile syringe and then centrifuged again on a discontinuous Percoll gradient to obtain clean spores, which were frozen in liquid nitrogen and stored at −80 °C [23].

### 2.3. cDNA Library Construction and PacBio SMRT Sequencing

The experimental process of full-length transcript sequencing includes sample testing, library construction, and sequencing on the sequencer. (1) The total RNA of *V. ceranae* spores was extracted using a SMARTer™ PCR cDNA Synthesis Kit; (2) synthesis of full-length cDNA was performed with a SMARTer™ PCR cDNA Synthesis Kit (TaKaRa, Japan); (3) BluePippin was used to screen full-length cDNA fragments, followed by construction of cDNA libraries of different sizes; (4) the full-length cDNA was amplified by PCR again; (5) end repair of full-length cDNA, followed by connection with SMRT dumbbell type connector and exonuclease digestion; and (6) BluePippin was used for secondary screening to obtain sequencing library. The quality of the library was tested by advanced molecular biology equipment (Thermofisher Scientific, Qubitmm 3 Fluorometer). Sequencing was performed only when the test results met the requirements. The PacBio SMRT sequencing of the constructed library of *V. ceranae* was conducted by Beijing Biomarker Technologies Co., Ltd. (Beijing, China).

### 2.4. cDNA Library Construction and Illumina Sequencing

(1) The total RNA was isolated from *V. ceranae* spores using a Trizol Kit (Thermo Fisher, Shanghai, China). (2) Oligo (dT) primers were used to isolate poly-A mRNA, followed by fragmentation and reverse transcription with random primers (Qiagen, Dusseldorf, Germany). Second-strand cDNAs were synthesized using RNase H and DNA polymerase I. The double-strand cDNAs were then purified using the QiaQuick PCR extraction kit (Qiagen, Dusseldorf, Germany). (3) After agarose gel electrophoresis, the required fragments were purified using a DNA extraction kit (Qiagen, Dusseldorf, Germany) and then enriched via PCR amplification in a total volume of 50 μL containing 3 μL of NEB Next USER Enzyme (NEB, Ipswich, MA, USA), 25 μL of NEB Next High-Fidelity PCR Master Mix (2×) (NEB, USA), 1 μL of Universal PCR Primer (25 mmol) (NEB, Ipswich, MA, USA), and 1 μL of Index (X) Primer (25 mmol) (NEB, Ipswich, MA, USA). The reaction conditions were set as follows: 98 °C for 30 s, followed by 13 cycles of 98 °C for 10 s, 65 °C for 75 s, and 65 °C for 5 s. (4) The amplified fragments were sequenced on the Illumina HiSeq 4000 platform (Illumina, San Diego, CA, USA) by Gene Denovo Biotechnology Co. (Guangzhou, China) following the manufacturer’s protocols.

### 2.5. Quality Control and Full-Length Transcript Identification

Based on the criteria of full passes ≥ 3 and sequence accuracy greater than 0.9, Circular Consensus (CCS) read sequences were extracted from the raw sequences and then corrected. Sequences were classified into full-length (FL) reads (including 5′ primer, 3′ primer, and polyA tail) and non-full-length (nFL) reads by detecting whether the CCS sequences contain the correct 5′ primer, 3′ primer, and polyA tail. The cDNA primer sequences and polyA sequences in the CCS reads were removed to obtain the inserted sequences during library construction. At the same time, the direction of strand synthesis was determined based on the differences in the primers at both ends during library construction, and the sequences were classified into FL reads, nFL reads, chimeric sequences, and non-chimeric sequences. The length of the FL reads reflects the length of the cDNA sequence during library construction, and the quality of library construction can be evaluated by statistical analysis of the length of the FL reads. Utilizing the IsoSeq module in the SMRTLink (v10.1) software [24], similar sequences within the FLNC reads, which were multiple copies of the same transcript, were clustered into a single cluster. Each cluster then generated a consensus isoform. Considering the high-quality screened sequences may be nFL reads due to the loss of 5′ end sequences during library preparation, the sequences that only differed in their 5′ terminal exons while having consistent exon sequences in the rest of the transcript were merged, and the longest sequence among these was regarded as the final transcript sequence.

To improve the accuracy of PacBio reads, the nFL reads were used to polish the above-obtained cluster consensus isoforms by SMRT Analysis 1.4 (https://investor.pacificbiosciences.com/news-releases/news-release-details/pacific-biosciences-software-upgrade-enhances-de-novo-genome) to obtain the FL-polished high-quality consensus sequences (accuracy ≥ 99%). Further, the low-quality isoforms were corrected using Illumina short reads obtained from the same samples using the LoRDEC tool (version 0.8) (Salmela and Rivals, 2014). According to the evaluation results of sample contamination, the reads obtained from Illumina sequencing that could be aligned with the NT database accounted for 33.17% of the total extracted reads. Among these, the reads aligned with *V. ceranae* accounted for 98.46% of the reads aligned with the NT database. No abnormal alignment was found in the alignment results.

### 2.6. Genomic Mapping

The corrected, high-quality CCS read was then mapped to the reference genome of *V. ceranae* (ASM98816v1) using the Genomic Mapping and Alignment Program (GMAP) [14,25]. The cDNA_Cupcake software (https://github.com/Magdoll/cDNA_Cupcake/wiki) was used to de-redundant the above alignment results, filter sequences with a consistency of less than 0.9 and coverage of less than 0.85, merge the alignment results with only differences in the 5′ end, and finally obtain the non-redundant full-length transcript sequence. The integrity of the non-redundant transcriptome was evaluated using BUSCO [26].

### 2.7. Annotation of Full-Length Transcripts

By using the BLAST tool (https://blast.ncbi.nlm.nih.gov/Blast.cgi), the identified full-length transcripts were, respectively, aligned to the Nr (ftp://ftp.ncbi.nlm.nih.gov/blast/db), KOG (http://www.ncbi.nlm.gov/KOG), eggNOG (http://eggnogdb.embl.de/#/app/emapper), GO (http://geneontology.org), KEGG (https://www.kegg.jp/kegg/), Swiss-Prot (http://www.ebi.ac.uk/swissprot/), and Pfam (http://pfam-legacy.xfam.org/) databases to gain corresponding annotations.

### 2.8. Analysis and Validation of AS Events

In general, there are five main types of AS events, including exon skipping (ES), intron retention (IR), alternative 5′ splice site (A5S), alternative 3′ splice site (A3S), and mutually exclusive exon (MEE). Following the method described by Foissac et al. the Astalavista software was employed to identify the AS events of the *V. ceranae* full-length transcripts with the default parameters, and then the types of AS events and the numbers of various types were calculated and presented as pie charts using the relevant tool in the BMKCloud platform (https://www.biocloud.net/) [27].

### 2.9. Structural Optimization of Annotated Genes in the Reference Genome

The full-length transcript obtained in this work with the annotated transcript on the reference genome of *V. ceranae* (assembly ASM98816v1) using the Gffcompare [28] software (http://ccb.jhu.edu/software/stringtie/gffcompare.shtml) to identify the regions outside the original gene boundaries that have continued alignment sequences, thus extending the 5′ UTR or (and) 3′ UTR of the gene, and modify the gene structure through the transcript.

### 2.10. Identification of lncRNAs

The prediction of lncRNA in novel transcripts was conducted by combining the most widely used coding potential analysis methods, which mainly included Coding Potential Calculator (CPC) analysis [29], Coding-Non-Coding Index (CNCI) analysis [30], Pfam protein domain analysis, and Coding-Potential Assessment Tool (CPAT) analysis [31]. CNCI (version 2) and CPC [30] (http://cpc.cbi.pku.edu.cn/) were used to evaluate the protein-coding potential of novel isoforms and new isoforms by default parameters. Meanwhile, isoforms were mapped to the SwissProt database to assess protein annotation. The intersection of both non-protein-coding potential results and non-protein annotation results was regarded as a candidate for long non-coding RNAs (lncRNAs). To better annotate lncRNAs at the evolution level, Infernal [32] (infernal.janelia.org) was used to assess the secondary structures and sequence conservation of lncRNAs. Cuffcompare software [33] was used to select the different types of lncRNAs, including lincRNA, intronic lncRNA, and antisense lncRNA. Fragments per kilobase of transcript per million mapped reads (FPKM) of both lncRNAs and mRNAs were calculated using StringTie (1.3.1) [34]. The transcript lengths, exon numbers and lengths, intron lengths, GC content, expression levels, and alternative splicing (AS) event numbers of lncRNAs were compared with those of mRNAs. The full names of all abbreviations used in this study are presented in Table 1.

## 3. Results

### 3.1. Quality Control and Identification of Full-Length Transcripts

**Figure 1:**
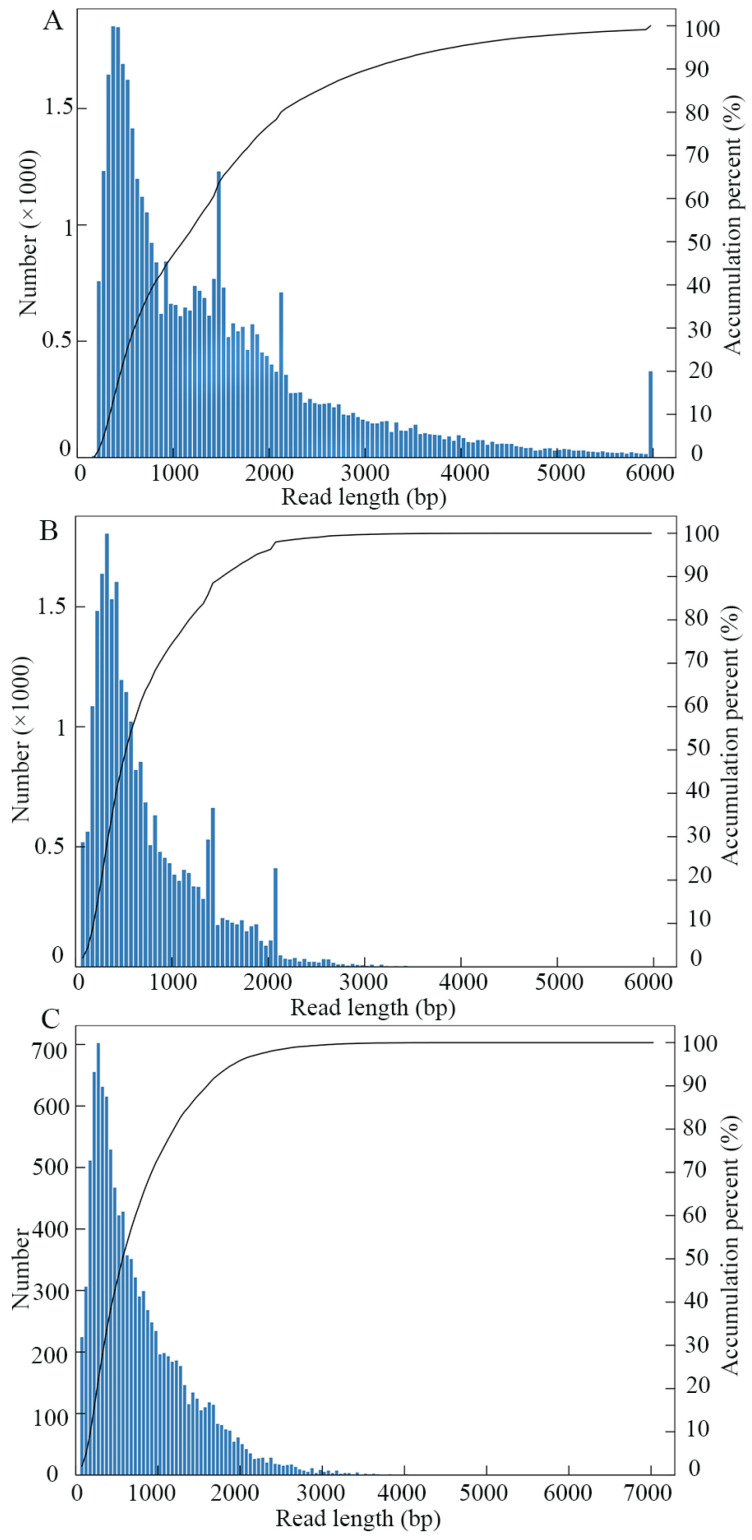
Length distribution of PcBio SMRT-generated long reads. (**A**–**C**) Number and length distribution of CCS, FLNC reads, and consensus isoforms.

Here, a total of 41,950 CCS were generated from PacBio SMRT sequencing (Table 2, Figure 1A). Among these, 25,068 were identified as FLNC reads (Table 2, Figure 1B). In addition, 10,900 consensus isoforms with an average length of 775 bp were obtained (Table 2, Figure 1C), and after polishing, 4387 high-quality, full-length transcripts were gained, including 699 novel transcripts (see Table S1 for details) that are not annotated in the reference genome of *V. ceranae*.

### 3.2. Functional Annotation of V. ceranae Full-Length Transcripts

Among the identified full-length transcripts, 778 (5.6%), 2083 (15%), 1202 (8.6%), 1559 (11.2%), 1457 (10.5%), 1232 (8.9%), 1702 (12.2%), and 3896 (28%) could be annotated to COG, GO, KEGG, KOG, Pfam, Swiss-Prot, eggNOG, and Nr databases, respectively. A total of 495 full-length transcripts could be simultaneously annotated in the above eight databases.

**Figure 2:**
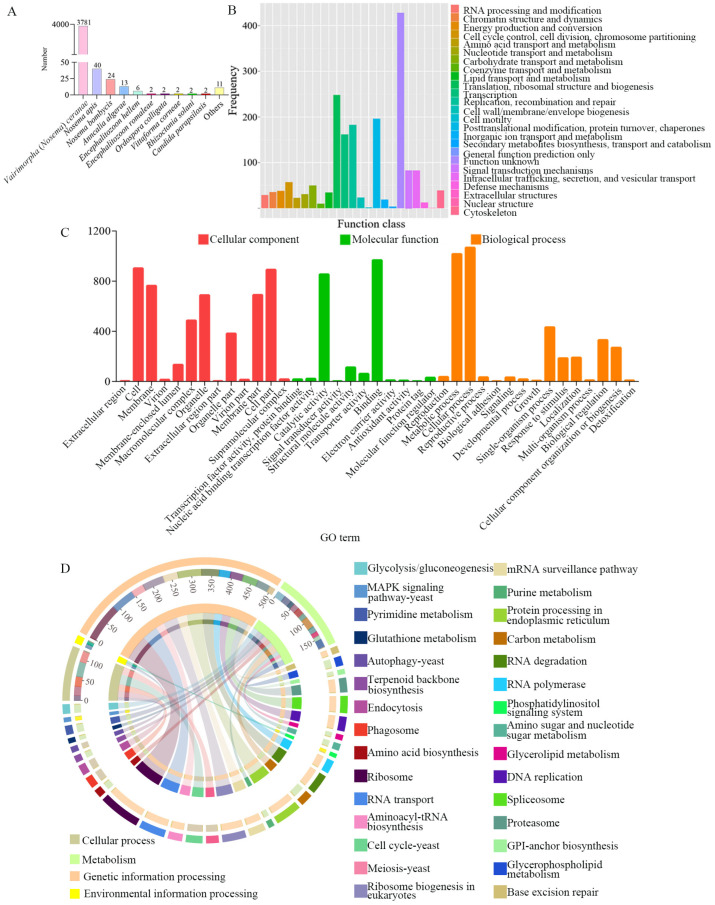
Annotation of *V. ceranae* full-length transcripts in the Nr (**A**), eggNOG (**B**), GO (**C**), and KEGG (**D**) databases.

According to annotations in the Nr database, the species with the largest number of annotated full-length transcripts was *V. ceranae* (97.37%), followed by *Nosema apis* (1.03%) and *Nosema bombycis* (0.62%) (Figure 2A). In the eggNOG database, the top three functional categories with the highest number of annotated full-length transcripts were (1) translation, ribosomal structure, and biogenesis; (2) posttranslational modification, protein turnover, and chaperones; and (3) replication, recombination, and repair (Figure 2B). As shown in Figure 2C, it is detected that 2083 full-length transcripts were involved in 39 GO terms in the GO database, including 15 terms related to biological processes such as cellular processes (1066) and metabolic processes (1015), 13 terms relevant to cellular components such as cells (901) and cell parts (890), and 11 terms relative to molecular functions like binding (1015) and catalytic activity (853). Additionally, 1202 full-length transcripts were associated with 71 KEGG pathways, such as the ribosome (91), protein processing in the endoplasmic reticulum (58), and RNA transport (57) (Figure 2D).

### 3.3. Analysis of Novel Transcripts Related to V. ceranae Virulence Factors and Energy Metabolism

On the basis of the annotations derived from the KEGG, Swiss-Prot, and Pfam databases, 19 full-length transcripts were detected to be associated with virulence factors such as spore wall protein (PB.414.3) and chitin synthase (PB.161.1, PB.1296.3). Additionally, 37 full-length transcripts relative to *V. ceranae* energy metabolism-associated pathways, such as glycolysis/gluconeogenesis as well as oxidative phosphorylation, were discovered (Table 3).

### 3.4. Identification of lncRNAs

**Figure 3:**
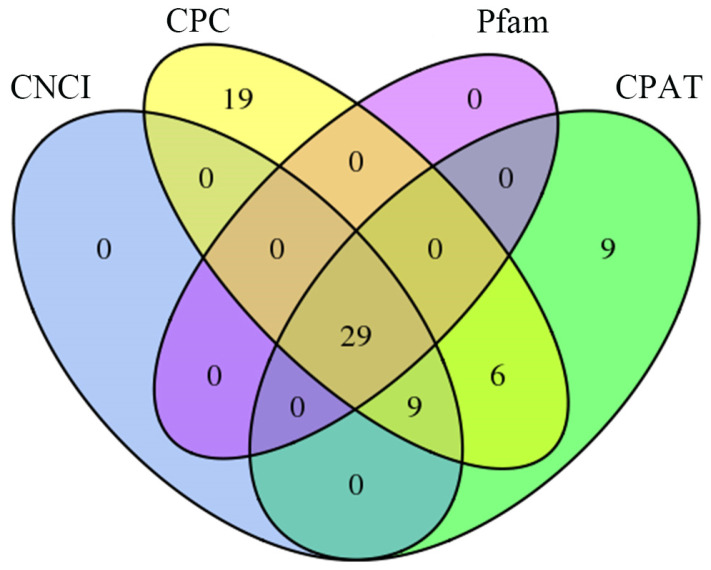
Venn Diagram of lncRNAs predicted by four different software programs.

**Figure 4:**
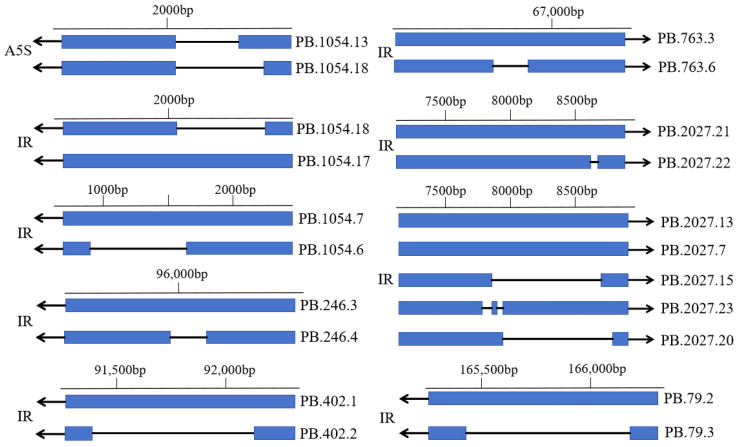
Schematic diagrams of A5S and IR events of *V. ceranae* genes. Transcript ID starting with PB represents the corresponding newly predicted full-length transcript by PacBio SMRT sequencing in this study. The transcripts in the reference genome of *V. ceranae* are, respectively, marked with dark blue; the gray line and numbers above indicate the genic region.

Here, 38, 63, 29, and 53 lncRNAs were, respectively, predicted by using CNCI, CPC, Pfam, and CPAT (Figure 3). After removing redundant lncRNAs, a total of 29 lncRNAs with high confidence were identified, including 12 sense-lncRNAs, 10 lincRNAs, and 7 antisense-lncRNAs (Figure 4, see Table S2 for details).

### 3.5. Structural Optimization of Annotated Genes on the V. ceranae Reference Genome

Based on the constructed full-length transcriptome, the structures of 225 genes annotated in the reference genome of *V. ceranae* were optimized (Table S3). In detail, the 5′ UTR of 90 genes was prolonged by 1~2098 bp, while the 3′ UTR of 106 genes was prolonged by 1~2473 bp (Table 4). In addition, the 5′ UTR and 3′ UTR of 29 genes were simultaneously prolonged (Table 4). Detailed information about the structural optimization of the above-mentioned 225 annotated genes is shown in Table S3.

### 3.6. Analysis of AS Events of V. ceranae Genes

Here, 11 AS events occurred in 6 genes, including 10 IR and 1 A5S (Figure 4; see also Table 5). Only one *V. ceranae* gene (GENE-AAJ76_2600001) was identified to contain a single APA site. Detailed information about the aforementioned 11 AS events is shown in Table 5.

## 4. Discussion

Though PacBio SMRT sequencing technology has been applied in the study of full-length transcriptomes of several fungi like *Verticillium dahlia* [35] and *Castanopsis carlesii* [36], relevant investigations of the *V. ceranae* full-length transcriptome have not yet been reported. In this work, based on PacBio SMRT sequencing of the *V. ceranae* spore sample, a total of 4387 non-redundant full-length transcripts were discovered, much more than the annotated transcripts (3265) in the current reference genome of *V. ceranae* (assembly ASM98816v1). This indicates that AS is a common phenomenon for such a single-cell fungal pathogen as *V. ceranae*. The identified full-length transcripts could provide a valuable supplement for the annotation of transcripts in the *V. ceranae* genome. A total of 2195 gene loci were identified, of which 848 were novel gene loci, indicating that the purified dormant spores still possessed abundant life activities. Further database annotation showed that 778, 2083, 1202, 1559, 1457, 1232, 1702, and 3896 full-length transcripts were annotated in COG, GO, KEGG, KOG, Pfam, Swiss-Prot, eggNOG, and Nr databases, respectively. The functional annotation of these full-length transcripts can offer a beneficial reference for subsequent functional research. In summary, the constructed full-length transcriptome of *V. ceranae* with functional annotation could provide a valuable resource for further studies.

When *V. ceranae* infects the host, it first squeezes the polar filament out of the spore wall, shoots it at the cell from a distance, and then injects the spore plasm into the cell [37]. In this current work, we identified a novel transcript associated with the infection apparatus, designated as PB.414.3, which encoded a spore wall protein. Microsporidium needs a constant supply of energy and substances to survive and thrive in host cells. On the one hand, microsporidia convert glucose into pyruvate through the glycolysis/gluconeogenesis pathway, and on the other hand, microsporidia steal energy and substances synthesized by the host for its own energy needs through the ATP/ADP translocator and ABC transporter [38,39]. Here, three novel transcripts associated with glycolysis/gluconeogenesis, such as PB.294.1, PB.293.1, PB.780.1, and PB.1959.3, were detected. In addition, we discovered four novel transcripts related to the ATP/ADP carrier protein (PB.677.1, PB.150.1, PB.1292.3, and PB.1293.1) and one novel transcript (PB.1301.8) associated with the ABC transporter. Whether and how these novel transcripts relevant to infection apparatus, virulence factor, and energy metabolism affect the infection process of *V. ceranae* is an attractive scientific question deserving further investigation. Recently, our group has established an RNAi-based platform for silencing various isoforms of the same honeybee gene. It is urgent to develop the technical route for functional study on the *V. ceranae* gene’s isoforms; thus, the molecular mechanism underlying the fungal infection could be illustrated at the isoform level. These newly discovered transcripts can serve as potential molecular targets for the development of anti-*V. ceranae* strategies and the control of bee nosemosis. LncRNAs are RNA molecules longer than 200 nucleotides that do not have protein-coding potential [40]. In recent years, lncRNAs have been recognized as pivotal regulators in numerous biological processes across a range of organisms. Functioning as powerful regulators, natural miRNA sponges, chromatin architecture modifiers, and transport vehicles for protein relocalization, they underscore the critical role lncRNAs play in gene expression and cellular functionality [41]. In this study, a total of 29 lncRNAs from *V. ceranae* were identified, including 12 sense lncRNAs, 10 lincRNAs, and 7 antisense lncRNAs. In previous work, following next-generation sequencing of *V. ceranae* spores, 59 antisense-lncRNAs, 21 lincRNAs, and 3 sense-lncRNAs were identified [42]. Intriguingly, intronic lncRNA was not detected by both PacBio SMRT sequencing and Illumina sequencing, collectively suggestive of a lack of this kind of AS event in *V. ceranae*, a unique fungus with an extremely reduced genome where introns are few [43]. 

Long-read data generated by PacBio SMRT sequencing can be utilized for optimization of the genomic annotation, as verified by relative studies on an array of animals, plants, and microorganisms, such as *S. scrofa* [19], *Juglans mandshurica* [44], and *A. apis* [20]. The 5′ and 3′ untranslated regions (UTRs) of genes are crucial binding sites for various regulators. The 5′ UTR influences ribosomal loading and promoter selection, thereby regulating translation efficiency [45]. The 3′ UTR can modulate mRNA’s translational activity or repression through interactions with miRNAs and RNA-binding proteins [46]. Here, on the basis of the identified full-length transcripts, the structures of 225 genes from the *V. ceranae* reference genome were optimized, accounting for 5.1% of total annotated genes. Specifically, 90 genes were extended at the 5′ UTR, 106 genes were extended at the 3′ UTR, and 29 genes were extended at both the 5′ UTR and 3′ UTR. The structural optimization of the 5′ UTR and 3′ UTR holds great significance for advancing the study of gene expression regulation in *V. ceranae*.

AS refers to the process whereby a gene’s precursor mRNA (pre-mRNA) is spliced in different ways to produce various mRNA splice isoforms, ultimately leading to protein products with diverse and sometimes antagonistic functional and structural characteristics [47]. This variability can result in distinct phenotypes within the same cell due to differences in expression levels [48]. AS significantly enhances the transcriptomic and proteomic diversity of a cell, a tissue, or an organism, enabling substantial alterations in the composition of gene transcription products without a corresponding increase in gene numbers [49]. Here, based on high-quality long-read data obtained from PacBio SMRT sequencing, we identified 11 AS events across six genes. In preliminary research, utilizing nanopore sequencing technology, we identified five AS events in five genes of *V. ceranae* [50]. The results together demonstrate that, as compared with nanopore long-read sequencing, PacBio SMRT sequencing has more advantages in structural detection and analysis, like AS event identification, which has been confirmed in many other species such as *Arabidopsis* [51], humans, and non-human primates [52].

## 5. Conclusions

In conclusion, the full-length transcriptome of *V. ceranae* was constructed and annotated utilizing PacBio SMRT sequencing; the structures of 225 annotated genes in the *V. ceranae* reference genome were optimized; abundant novel genes, transcripts, and lncRNAs were discovered; and 10 IR as well as 1 A5S were identified. Our findings provide a valuable resource and a vital basis for further molecular and omics research on *N. ceranae*.

## Figures and Tables

**Table 1:** The list of full names of all abbreviations used in this study.

Full Names	Abbreviations
Single-molecule real-time	SMRT
Circular consensus	CCS
Full-length non-chimeric	FLNC
alternative splicing	AS
Next-generation sequencing	NGS
Third generation sequencing	TGS
Alternative polyadenylatio	APA
Full-length	FL
Non-full-length	nFL
Genomic Mapping and Alignment Program	GMAP
Exon skipping	ES
Intron retention	IR
Alternative 5′ splice site	A5S
Alternative 3′ splice site	A3S
Mutually exclusive exon	MEE
Potential Calculator	CPC
Coding-Non-Coding Index	CNCI
Coding-Potential Assessment Tool	CPAT

**Table 2:** Summary of long reads produced from PacBio SMRT.

	Number
CCS	41,950
Read bases of CCS	61,124,354
Mean read length of CCS	1457
Mean number of passes	95
Full-length non-chimeric reads	25,068
Full-length non-chimeric percentage	59.76%
Consensus isoforms	10,900
Average read length of consensus isoforms	775
Polished high-quality isoforms	10,900
Polished low-quality isoforms	0

**Table 3:** Summary of the full-length transcript relevant to *V. ceranae* virulence factors and energy metabolism.

	Annotation	Transcript ID
Virulence factors	ADP/ATP carrier protein	PB.150.1, PB.677.1, PB.1292.3, PB.1293.1
	Chitin synthase	PB.161.1, PB.1296.3
	Iron-sulfur clusters transporter	PB.1602.1, PB.1302.1
	ABC transporter	PB.54.5
	Iron-sulfur clusters transporter, ABC transporter	PB.1301.8
	Spore wall protein	PB.414.3
Energy metabolism	Glycolysis/Gluconeogenesis	PB.1959.3, PB.294.1, PB.293.1, PB.780.1, PB.1959.3
	Oxidative phosphorylation	PB.1107.1, PB.1797.6, PB.1608.1, PB.1608.2
	Frataxin	PB.1632.4

Note: Transcript ID starting with PB represents the corresponding newly predicted full-length transcript by PacBio SMRT sequencing in this study.

**Table 4:** Details of structural optimization of annotated genes in the *V. ceranae* reference genome (13 displayed only).

Gene ID	Locus	Strand	Site	Original Site	Optimize Site
gene-AAJ76_3600024363	NW_020169331.1:24363–25655	+	3′	25,376	25,655
gene-AAJ76_800027588	NW_020169303.1:26663–27588	−	5′	26,860	26,663
gene-AAJ76_1100030560	NW_020169306.1:30560–32315	+	3’	31,945	32,315
gene-AAJ76_1900029507	NW_020169314.1:29503–30905	+	5’	29,507	29,503
gene-AAJ76_1900029507	NW_020169314.1:29503–30905	+	3′	30,883	30,905
gene-AAJ76_1180002683	NW_020169413.1:1478–3041	−	3′	2683	3041
gene-AAJ76_2200042944	NW_020169317.1:42944–44008	+	3′	43,930	44,008
gene-AAJ76_1100054231	NW_020169306.1:54231–55342	+	3′	54,851	55,342
gene-AAJ76_200036678	NW_020169297.1:35879–36711	−	5′	35,890	35,879
gene-AAJ76_200036678	NW_020169297.1:35879–36711	−	3′	36,706	36,711
gene-AAJ76_100042163	NW_020169296.1:41211–42484	−	5′	41,228	41,211
gene-AAJ76_100042163	NW_020169296.1:41211–42484	−	3′	42,163	42,484
gene-AAJ76_580008628	NW_020169353.1:7727–8628	−	5′	8332	7727

**Table 5:** Details of 11 AS events of *V. ceranae* genes.

Type of AS Event	Genomic Location	Gene ID	Transcript ID
Alternative 5′ splice site	NW_020169316.1:348-2409C	PB.1054	PB.1054.18, PB.1054.13
Intron retention	NW_020169316.1:348-2409C	PB.1054	PB.1054.17, PB.1054.18
Intron retention	NW_020169316.1:348-2409C	PB.1054	PB.1054.7, PB.1054.6
Intron retention	NW_020169298.1:95363-96278C	PB.246	PB.246.3, PB.246.4
Intron retention	NW_020169300.1:91266-92414C	PB.402	PB.402.1, PB.402.2
Intron retention	NW_020169306.1:66670-67167W	PB.763	PB.763.3, PB.763.6
Intron retention	NW_020169296.1:164677-166306C	PB.79	PB.79.2, PB.79.3
Intron retention	NW_020169415.1:7382-8798W	PB.2027	PB.2027.21, PB.2027.22
Intron retention	NW_020169415.1:7382-8798W	PB.2027	PB.2027.13/PB.2027.7, PB.2027.15
Intron retention	NW_020169415.1:7382-8798W	PB.2027	PB.2027.13/PB.2027.7, PB.2027.23
Intron retention	NW_020169415.1:7382-8798W	PB.2027	PB.2027.13/PB.2027.7, PB.2027.20

Table note: Transcript ID starting with PB represents the corresponding newly predicted full-length transcript by PacBio SMRT sequencing in this study.

## Data Availability

All the data are contained within the article.

## Supplementary Materials

The following supporting information can be downloaded at: https://www.mdpi.com/article/10.3390/genes15091111/s1, Table S1: Details of 699 novel transcripts annotated in the *V. ceranae* reference genome; Table S2: Detailed information about 29 non-redundant lncRNAs in *V. ceranae*; Table S3: Details of structural optimization of 225 annotated genes in the *V. ceranae* reference genome.
